# Echocardiography of isolated subacute left heart tamponade in a patient with cor pulmonale and circumferential pericardial effusion

**DOI:** 10.1186/1476-7120-8-27

**Published:** 2010-07-14

**Authors:** Tomaž Marš, Helena Mikolavčič, Barbara Salobir, Matej Podbregar

**Affiliations:** 1 Institute of Pathophysiology, Faculty of Medicine, University of Ljubljana, Slovenia; 2 Clinical Department for Internal Intensive Care, University Medical Center Ljubljana, Slovenia; 3 Clinical Department for Pulmonary Disease and Alergology, University Medical Center Ljubljana, Slovenia

## Abstract

Patients with advanced idiopathic pulmonary artery hypertension have often a chronic pericardial effusion. It is the result of increased transudation and impaired re-absorption due to elevated venous pressure. These patients have pre-existent symptoms and signs of chronic right heart failure. High degree of suspicion is required to detect of development of an atypical form of tamponade with isolated compression of left heart chambers as shown in present case report. Transthoracic echocardiography provides a rapid access to the correct diagnosis, a prompt relief of symptoms following the ultrasound guided pericardiocentesis and important diagnostic tool for regular follow up of patients thereafter as shown in our case report.

## Background

Circumferential pericardial effusion typically results in biventricular tamponade and equalization of intracardiac and pericardial pressure during diastole. In classic subacute tamponade the rising of pericardial pressure causes a progressive collapse of right atrium and ventricle preventing venous return to the right atrium. Symptoms and signs referable to increased filling pressure and diminished cardiac output ensue. Patients presents with dyspnea, orthopnea, peripheral edema, fatigability, hepatic engorgement. The three principal features: jugular venous distention, soft or absent heart sounds and hypotension (Beck's triad), tachycardia and pulsus paradoxus are present. However, tamponade may involve the right or left heart. While isolated left ventricular tamponade can occur as a postoperative complication form localized posterior pericardial effusions or hematoma, circumferential pericardial effusions leading to left heart tamponade are rare [1].

## Case presentation

We present a clinical course and echocardiographic examination in a patient with idiopathic pulmonary artery hypertension (IPAH) and chronic pericardial effusion who developed a subacute isolated left heart tamponade.

**Figure 1:**
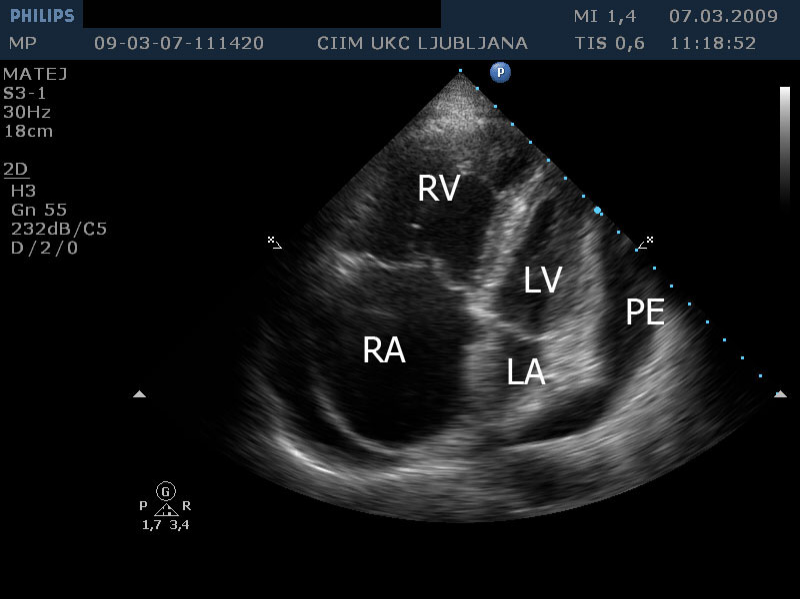
**Transthoracic echocardiography from apical four chamber view shows enlarged right ventricle (RV) and right atrium (RA)**. There is pericardial effusion (PE) compressing left ventricle (LV) and left atrium (LA).

**Figure 2:**
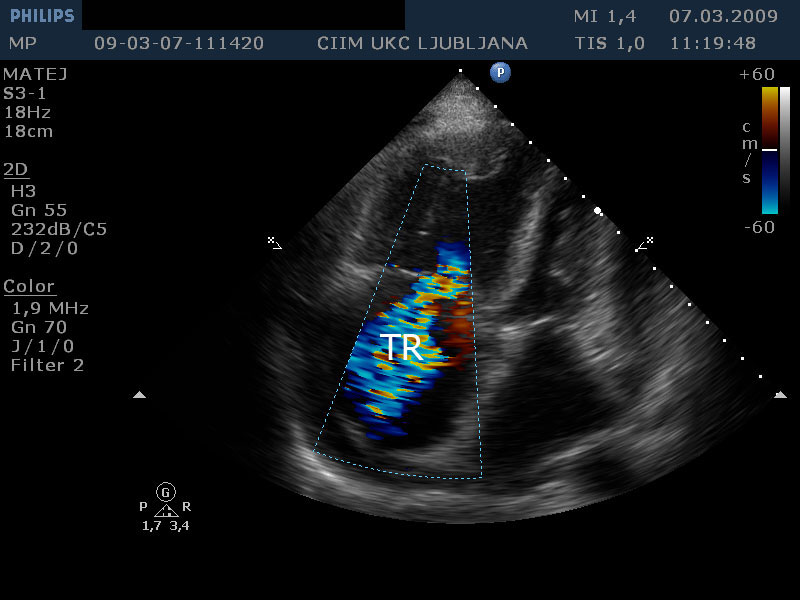
**Transthoracic echocardiography from apical four chamber view shows massive tricuspid regurgitation (TR)**.

**Figure 3:**
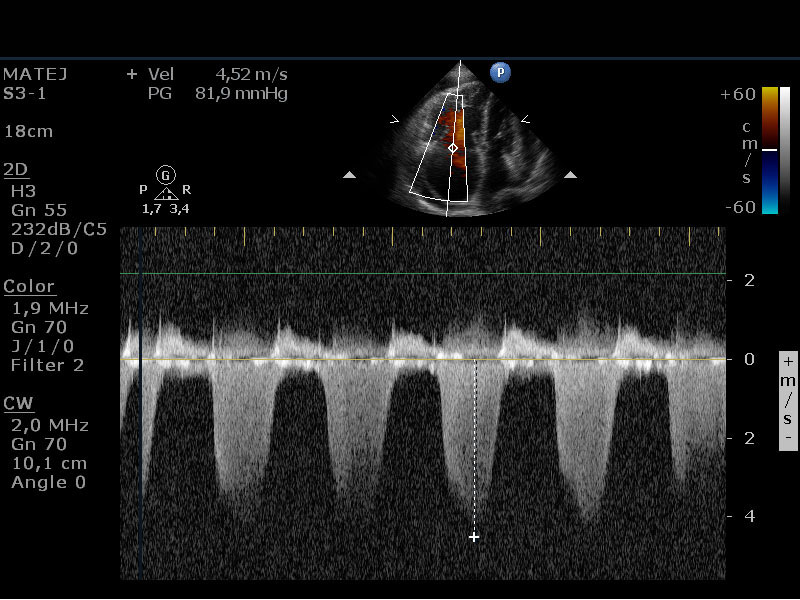
**Right ventricular (RV) pressure was estimated by measuring tricuspid regurgitation (TR) velocity as stated in the Bernoulli equation: RV pressure = 4 × (tricuspid regurgitation velocity)^2 ^+ central venous pressure**.

**Figure 4:**
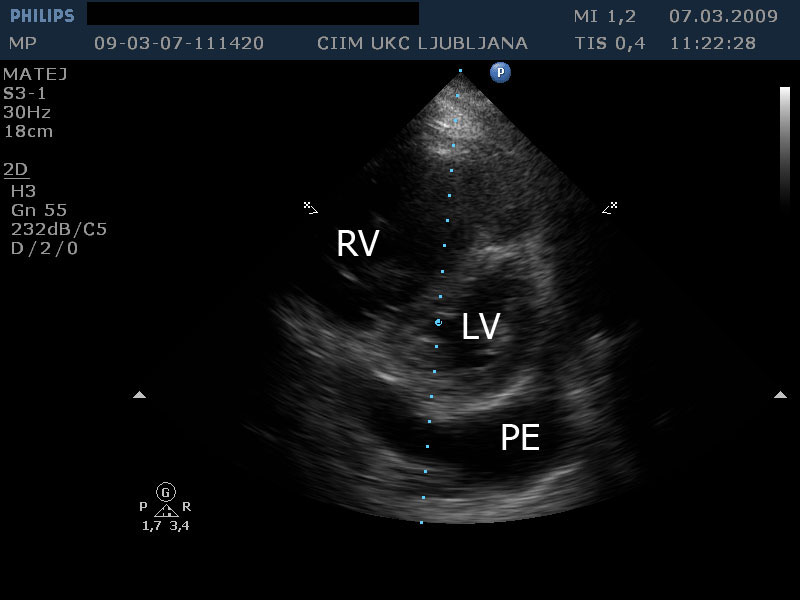
**Transthoracic echocardiography from short-axis plane at the papillary muscle level shows enlarged right ventricle (RV) with paradoxical movement of intraventricular septum**. Left ventricle is compressed by RV and pericardial effusion (PE).

A 57-year-old patient with IPAH and a previously known chronic pericardial effusion presented in an outpatient clinic with symptoms of dyspnea on exertion, in the last days even at rest, ortopnea and leg edema. During the past few months he was in good physical condition. He was on therapy with sildenafil, amlodipine, acenocoumarol and had a combination inhaler containing fluticasone propionate and salmeterol xinafoate. The clinical examination showed distended jugular veins, leg edema and an accentuated second heart sound. A chest radiogram showed an enlarged heart shadow. The echocardiographic examination showed a dilated right atrium and ventricle (Figure 1) with reduced ejection fraction, severe tricuspid insufficiency (Figure 2), systolic right ventricular pressure 82 mm Hg plus central venous pressure (Figure 3), the inferior vena cava larger than 2.5 cm with no respiration variability. Clinically estimated central venous pressure was approximately 20 mm Hg. Compared with the previous transthoracic echocardiographic examination, there was enlarged pericardial effusion, 3 cm behind the left ventricular posterior (Figure 4) wall with diastolic collapse of left atrium and ventricle (Additional files 1, 2 and 3).

After the normalisation of the haemosthasis with fresh frozen plasma echocardiographicly guided pericardiocentesis using a parsternal approach was performed. Immediately after removal of 250 ml of yellow fluid the patient became normopnoic. There was still 1.5 cm large pericardial effusion behind the left ventricular posterior wall without left heart collapse (Additional file 4).

The pericardial fluid was a borderline transudate with slightly elevated proteins. Cytological studies for malignancy, microbiological microscopic examination and cultures (included Mycobacterium tuberculosis) were negative. Rheumatic markers (antinuclear antibodies, anti-double-strand DNA, anticardiolipin antibodies, lupus anticoagulants, rheumatoid factor), angiotensin-convertyng enzyme and tumor markers (tissue polypeptide antigen, neuron-specific enolase, alpha-fetoprotein, prostate-specific antigen, carcinoembryonic antigen) were all in normal range. The function of the thyroid gland was normal.

## Discussion

Patients with cor pulmonale and circumferential pericardial effusion develop an atypical form of cadiac tamponade with isolated left heart compression. Pre-existing pulmonary arterial hypertension can modify the classic presentation. Symptoms and signs of right heart failure could already be present, so a high index of suspicion for tamponade is required in every worsening of right heart failure symptoms. When the pericardial pressure starts to increase in a patent with cor pulmonale, elevated pressure in right heart chambers prevent right atrial and ventricular compression, but while the pericardial pressure rises to the point to exceed left chambers pressure, this results first in diastolic collapse of left atrium and later on in left ventricle collapse due to a transient reversal of the transmural pressure [2,3]. Signs of impaired filling of left ventricle ensue leading to a drop in cardiac output.

The most probable mechanism of accumulation of pericardial fluid in patients with IPAH is transudation and impaired re-absorption of pericardial fluid due to elevated venous hydrostatic pressure in the setting of cor pulmonale.

In the setting of pulmonary arterial hypertension large hemodynamically significant pericardial effusions might be treated surgically and/or conservative and it is known that prognosis of patients with this complication is poor [4,5]. However, our patient has been stable throughout one year period after pericardiocentesis on his regular therapy after titration of diuretic furosemide (one tablets of 40 mg two to four times weekly) according to signs of right heart failure and measurements of NT-pro BNP (NT-proBNP before 600 ng/L, at the time of detection 1029 ng/L and one year after detection of circumferential pericardial effusion 601 ng/L). Additional regular repeated echocardiographic examinations were performed.

The right ventricle-to-right atrial pressure gradient may be difficult to estimate in the setting of severe tricuspid regurgitation, when there is a large color flow regurgitant jet. In this case, the peak velocity may not reflect the true pressure gradient.

In conclusion, patients with advanced IPAH have often a chronic pericardial effusion. It is the result of increased transudation and impaired re-absorption due to elevated venous pressure. These patients have pre-existent symptoms and signs of chronic right heart failure. High degree of suspicion is required to detect of development of an atypical form of tamponade with isolated compression of left heart chambers. Transthoracic echocardiography provides a rapid access to the correct diagnosis, a prompt relief of symptoms following the ultrasound guided pericardiocentesis and important diagnostic tool for regular follow up of patients thereafter.

## Consent

Written informed consent was obtained from the patient for publication of this case report and accompanying images. A copy of written consent is available for review by the Editor-in-Chief of this journal.

## Competing interests

The authors declare that they have no competing interests.

## Authors' contributions

TM: carried out interpretation and drafted the manuscript

HM: carried out interpretation and drafted the manuscript

BS: treated the patient, carried out interpretation and drafted the manuscript

MP: treated the patient, made acquisition of data, carried out interpretation and drafted the manuscript

All authors read and approved the final manuscript.

## Supplementary Material

Additional file 1 **Transthoracic echocardiography from apical four chamber view shows enlarged right ventricle (RV) and right atrium (RA)**. There is pericardial effusion (PE) compressing left ventricle (LV) and left atrium (LA). Click here for file

Additional file 2 **Transthoracic echocardiography from short-axis plane at the papillary muscle level shows enlarged right ventricle (RV) with paradoxical movement of intraventricular septum**. Left ventricle is compressed by RV and pericardial effusion (PE). Click here for file

Additional file 3 **Transthoracic echocardiography from apical four chamber view shows enlarged right ventricle (RV) and tricuspid regurgitation (TR)**. Click here for file

Additional file 4 **Transthoracic echocardiography from apical four chamber view after pericardiocentesis**. Right ventricle (RV), right atrium (RA), left ventricle (LV), left atrium (LA). Click here for file
